# Vascular patterns provide therapeutic targets in aggressive neuroblastic tumors

**DOI:** 10.18632/oncotarget.7661

**Published:** 2016-02-24

**Authors:** Irene Tadeo, Gloria Bueno, Ana P. Berbegall, M. Milagro Fernández-Carrobles, Victoria Castel, Marcial García-Rojo, Samuel Navarro, Rosa Noguera

**Affiliations:** ^1^ Pathology Department, Medical School, University of Valencia, INCLIVA, 46010 Valencia, Spain; ^2^ VISILAB, E.T.S. Ingenieros Industriales, University of Castilla-La Mancha, 13071 Ciudad Real, Spain; ^3^ Pediatric Oncology Unit, University and Polytechnic Hospital La Fe, 46026 Valencia, Spain; ^4^ Department of Pathology, Hospital de Jerez de la Frontera, 11407 Jerez de la Frontera, Cádiz, Spain

**Keywords:** extracellular matrix, blood vessels, capillaries, sinusoids, neuroblastoma

## Abstract

Angiogenesis is essential for tumor growth and metastasis, nevertheless, in NB, results between different studies on angiogenesis have yielded contradictory results. An image analysis tool was developed to characterize the density, size and shape of total blood vessels and vascular segments in 458 primary neuroblastic tumors contained in tissue microarrays. The results were correlated with clinical and biological features of known prognostic value and with risk of progression to establish histological vascular patterns associated with different degrees of malignancy. Total blood vessels were larger, more abundant and more irregularly-shaped in tumors of patients with associated poor prognostic factors than in the favorable cohort. Tumor capillaries were less abundant and sinusoids more abundant in the patient cohort with unfavorable prognostic factors. Additionally, size of post-capillaries & metarterioles as well as higher sinusoid density can be included as predictive factors for survival. These patterns may therefore help to provide more accurate pre-treatment risk stratification, and could provide candidate targets for novel therapies.

## INTRODUCTION

Neuroblastic tumors (NB) cause 15% of childhood deaths by cancer [1]. Despite the definition by the International NB Risk Group (INRG) of seven clinical and biological parameters that interplay to define a very low to high risk of progression [2], greater efforts are needed to enhance survival, especially in the high-risk group. In NB, results between different studies on angiogenesis are conflicting, some indicate a prognostic value and others reject such conclusions [3-8]. This discrepancy may be due to the different methods or materials utilized. Most of these studies focussed on microvessel density [3-6, 8], a few studies have described morphological changes detected subjectively [7] or have tried to distinguish between different types of vascularization based on integrin expression [9]. More robust morphometric techniques able to standardize measurements and analyze whole sets of immunohistochemical (IHC) images are needed to identify, describe and quantify blood vascularization alterations of the extracellular matrix (ECM). As in healthy tissues, the properties and composition of the vascular system may be associated with histological subtypes of NB and could therefore be morphologically and topologically characterized and linked to patient prognosis. This may then provide more accurate pre-treatment risk stratification or candidate elements for novel therapies. In order to determine histological patterns associated with different degrees of malignancy, we developed a novel and accurate image analysis algorithm, capable of quantifying and characterizing blood vessels, and performed statistical analyses to correlate the resulting variables with the INRG prognostic features and the risk of relapse or death.

## RESULTS

### Neuroblastic tumors may present vessel wall mosaicism and accompany immune response

**Figure 1:**
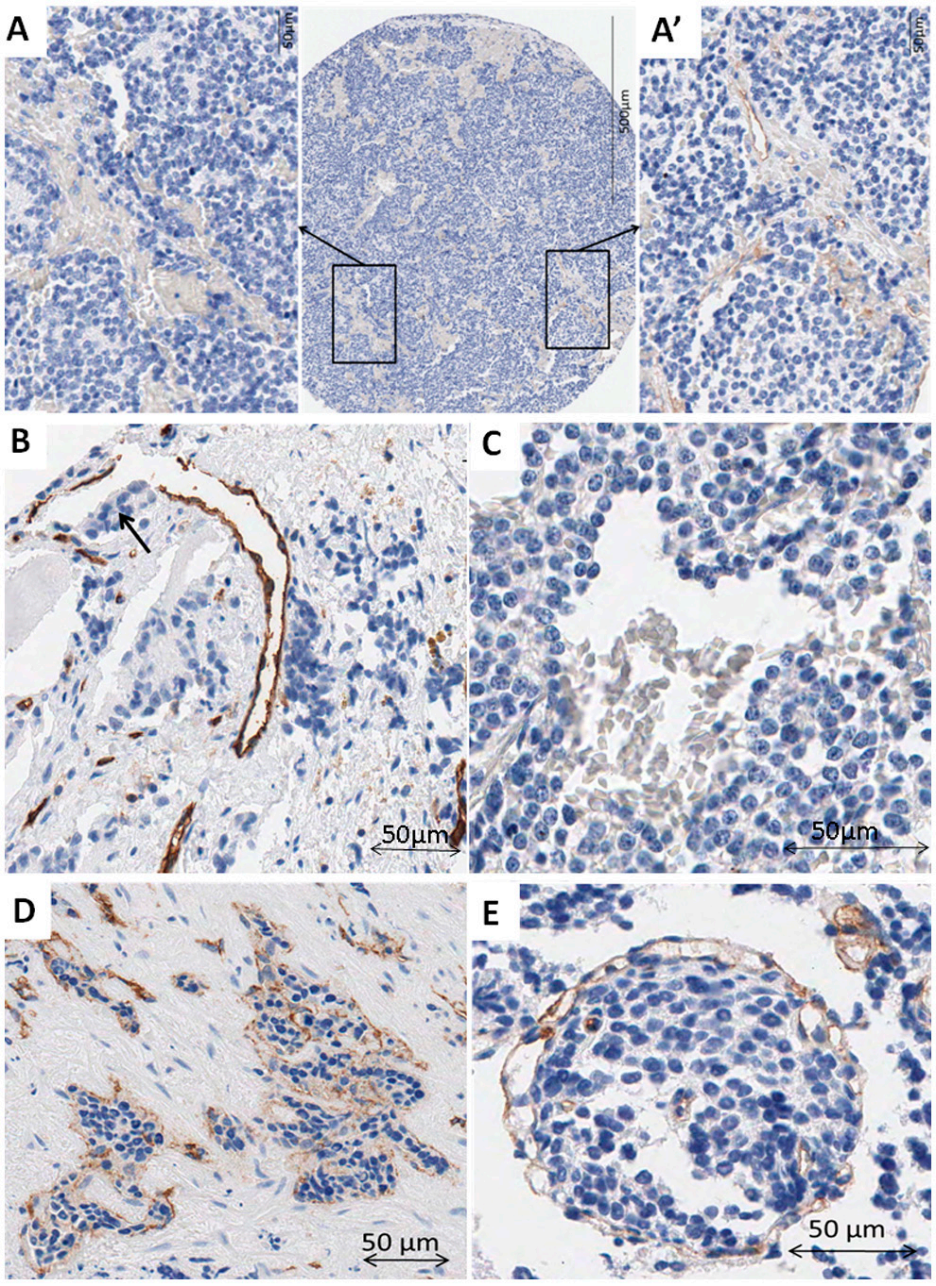
A–A') Example of a cylinder with no expression for CD31 antibody except for two stained blood vessels **A.** Detail of a negative area filled with erythrocytes. **A'.** Detail of an area with two CD31 positive blood vessels. **B.** Example of a blood vessel with incomplete outline. Note the neuroblastic-like cells occupying the area without epithelial cells (arrow). **C.** Example of a vascular structure outlined by cubic cells not expressing CD31, with erythrocytes inside. **D.** Blood vessel invaded by tumor cells. **E.** It is difficult to determine if tumor cells are invading a blood vessel or if many intermingled vessels surround a tumor cell cluster. Some small vessels can be observed inside the tumor cell cluster.

**Figure 2:**
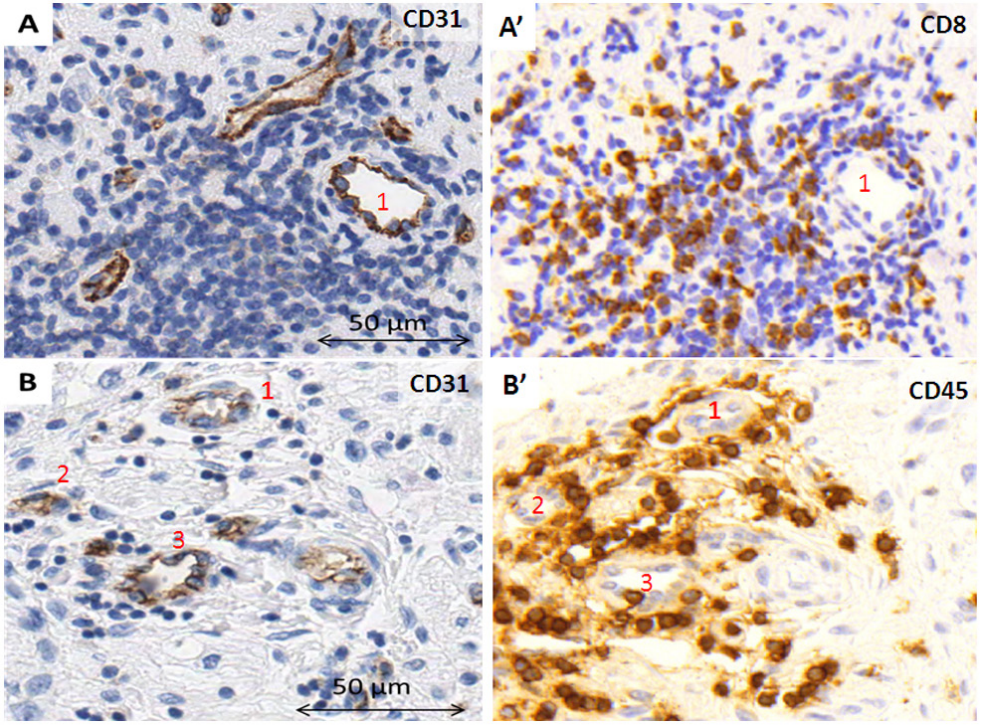
High endothelial venules **A.** within a tertiary lymph node, **B.** in close relationship with individual leukocytes within the tumor stroma. **A', B'.** Stainings to visualize the surrounding immune cells.

In some of the samples, lack of staining caused some vascular structures to remain undetected. Nevertheless, in few TMA cylinders, although most of the tissue area lacked CD31 expression, in what could be focal hemorrhages, one or two vessels with CD31 positive endothelial cells were found (Figure 1A, A'). Additionally, in some vessels, parts of the endothelial cell outline were not reactive to CD31, maybe constituting a vessel wall mosaicism, where both endothelial and tumor cells line the blood vessel (Figure 1B), or could correspond to an angiomatoid pattern, with vessels only covered by round or polygonal cells, which could be neuroblastic cells, immune cells or stem cells (Figure 1C). Additionally, vascular invasion was observed in NB (Figure 1D, 1E). In some cases, high endothelial venules (HEV) or activated post-capillaries were found associated to tertiary lymph nodes or to non-grouped leukocytes (Figure 2).

### Quantification of the blood vessels

CD31 reactivity was evaluable in 373 samples (81.4%) and positive in 325 samples (71%). CD31 was specific for blood vessels as shown in the Supplementary Figure S1. Mean blood vessel density was 161±177 vessels/mm^2^ occupying a stained area (SA) of 1.7%±1.2 of the tissue. Mean blood vessel dimensions were 14μm long × 7μm wide, with a perimeter of 42μm and an average area of 101μm^2^. All morphometric descriptors are provided in the Supplementary Table S1. The morphological changes quantified in the cohort are illustrated in Table 1.

**Table 1:** Schematic representation of the morphometric parameters measured and how the results are stated in the paper

Parameter	Low		High	
	Stated in the text	Schema	Stated in the text	Schema
Quantity				
Density	Low density	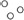	High density	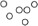
Stained area (SA)	Low SA	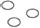	High SA	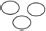
Relative density/SA of blood vessels corresponding to a given segment compared with the total vascularization	Low proportion of blood vessels	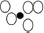	High proportion blood vessels	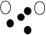
Size				
Area	Small	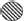	Large	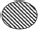
Length	Short	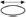	Long	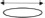
Width	Thin	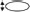	Thick	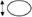
Perimeter	Small	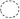	Large	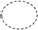
Shape				
Aspect^1^	Round	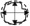	Ovoid	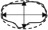
Roundness^2^*	Few protrusions	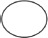	Abundant protrusions	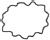
Perimeter ratio^3^*	Irregular outline	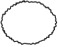	Regular outline	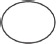
Deformity^4^*	Weak deformity	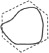	Strong deformity	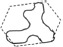
Shape factor^5^*	Similar to convex contour	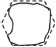	Dissimilar to convex contour	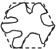
Branching	Unbranched	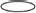	Branched	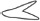

(1) Aspect: Major axis/minor axis (of ellipse equivalent to the vessel).

(2) Roundness: Perimeter^2^/(4*π*area).

(3) Perimeter ratio: Convex contour perimeter/perimeter (convex contour: outline of the polygon best-fitting to the real shape).

(4) Deformity: Convex contour area-area (μm^2^).

(5) Shape factor: Matches the shape (including the centroids, the area, and information about the orientation) of the vessels and the convex contour (formula: max*_i_*_=1,2_[│*m_i_*^A^-*m_i_*^B^│/│*m_i_*^A^│]; *m_i_*^A/B^=sign (*h_i_*^A/B^)· log(*h_i_*^A^); A/B=Hu moments of real contour/convex contour; *í* = seven Hu invariant).

* All these parameters provide information about the degree of deviation from a round and regular blood vessel, using different approaches.

### Unfavorable NB is associated with a high density of deformed blood vessels

**Figure 3:**
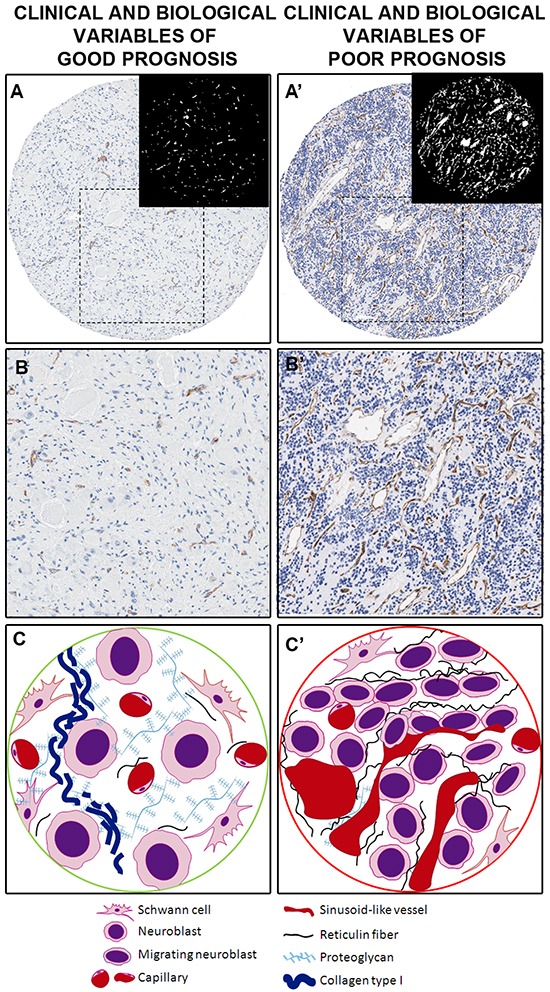
Examples and illustration of the vascular patterns in a neuroblastic tumor with favorable prognostic factors (A–C) and with unfavorable prognostic factors (A'–C'), illustrated by a GN and a pdNB, respectively **A.** Sample corresponding to a favorable NB. Differentiated histology with ganglion cells can be appreciated, with few blood vessels, mostly corresponding to capillaries. A binarized image is presented with blood vessels in white on a black background for a better view. **A'.** Sample corresponding to an unfavorable sample with rich blood vascularization, mostly corresponding to abnormal sinusoids. A binarized image is presented with blood vessels in white on a black background for a better view. **B.** and **B'.** Detail of the A and A' areas in the squares. **C.** Cylinder presenting a vascular pattern of a neuroblastic tumor with favorable prognostic factors where neuroblastic cells differentiate in a microenvironment well irrigated by capillaries, rich in fundamental substance to which they can bind and with a very loose meshwork of reticulin fibers. **C'.** Cylinder presenting a vascular pattern of a neuroblastic tumor with favorable prognostic factors presenting an extracellular matrix with a poor amount of capillaries and an increased presence of sinusoid vessels, which could promote cell extravasation and intravasation. A poorly-porous extracellular matrix is also defined by the reticulin fiber scaffolding.

NB with unfavorable histology presented a higher total blood vessel density that covered a higher SA than NB with favorable histology (Figure 3). The blood vessels of *MYCN* amplified (MNA) tumors and of those from high-risk patients were larger than the vessels of *MYCN* non-amplified (MNNA) tumors and from non-high-risk patients. Larger area was also associated with greater undifferentiation within the three NB histopathological categories. Blood vessels were rounder for most of the poor-prognostic categories of the INRG variables and therefore the high-risk group, compared with their favorable counterparts. NB blood vessels presented stronger deformity and more branching in MNA tumors and, in general, those tumors from high-risk patients. Table 2 provides the p-values and the nature of the relationship between the total vascularization and the INRG poor-prognostic factors.

**Table 2:** p-values and nature of the relationship between total vascularization and the INRG poor-prognostic factors

Parameter	Stage: metastatic	Age: >18 months	Histopathology: (pd, uNB)	*MYCN:* MNA	Genetic profile: SCA	11q: 11qD	Risk group: high-risk
Density	-	-	0.008↑	-	-	-	-
SA	-	-	0.033↑	-	-	-	-
Area	-	-	*0.027↑	0.000↑	-	-	0.006↑
Length	-	-	-	0.001↑	-	-	0.006↑
Width	-	-	-	0.000↑	-	-	0.000↑
Perimeter	-	-	-	0.000↑	-	-	0.006↑
Aspect	0.036↓	-	0.000↓	0.001↓	-	0.043↓	0.002↓
Roundness	-	-	-	-	-	-	-
Per. ratio	-	-	-	-	-	-	-
Deformity	-	-	-	0.001↑	-	-	0.026↑
Shape factor	-	-	0.014↓	0.046↓	-	-	-
Branching	0.045↑	-	-	0.000↑	-	-	0.001↑

SA: Stained area, Per. ratio: perimeter ratio, -: not statistically significant, ↑/↓: higher or lower median value for the poor-prognostic group(s)

* statistically significant differences between the different degrees of differentiation within the NB histological category.

### Unfavorable NB present mainly irregular sinusoid vessels, whereas favorable NB present mainly capillary networks

Capillary SA and relative SA were low in MNA tumors as well as in tumors from patients ≥18 months. Capillary relative density was also low in MNA tumors (Figure 3). Nevertheless, capillary density and SA were higher in the undifferentiated tumors compared to their favorable counterparts. Additionally, higher SA correlated with greater undifferentiation within the three NB histopathology categories. In general, the capillaries from tumors with INRG unfavorable features were larger, rounder, presented fewer protrusions and weaker deformity. They were also more similar to convex contour and more branched than their favorable counterparts. In contrast, discrepant results with respect to the parameters deformity and shape factor were found in tumors with unfavorable histology. Table 3 shows the p-values and the nature of the relationship between the capillaries and the INRG poor-prognostic factors.

**Table 3:** p-values and nature of the relationship between the capillaries and the INRG poor-prognostic factors

Parameter	Stage: metastatic	Age: >18 months	Histopathology: (pd, uNB)	*MYCN:* MNA	Genetic profile: SCA	11q: 11qD	Risk group: high-risk
Density	-	-	0.008↑	-	-	-	-
SA	-	0.040↓	0.028↑, *0.019↑	0.021↓	-	-	-
Rel. density	-	-	-	0.018↓	-	-	-
Rel. SA	-	0.039↓	-	0.032↓	-	-	-
Area	-	-	0.001↑	0.001↑	-	-	0.006↑
Length	-	-	0.010↑	-	-	-	-
Width	-	-	0.001↑	0.001↑	-	-	0.003↑
Perimeter	-	-	0.001↑	-	-	-	-
Aspect	-	-	0.001↓	0.000↓	-	-	0.001↓
Roundness	-	-	0.001↓	0.000↓	-	-	0.002↓
Per. ratio	-	-	-	-	-	-	-
Deformity	-	0.046↓	0.015↑	-	-	-	-
Shape factor	0.026↓	-	0.000↓	0.000↓	-	-	0.000↓
Branching	-	-	0.007↑	-	-	-	-

SA: Stained area Rel. density: relative density, Rel. SA: relative SA, Per. ratio: perimeter ratio, -: not statistically significant, ↑/↓: higher or lower median value for the poor-prognostic group(s)

* statistically significant differences between the different degrees of differentiation within the NB histological category.

Highest sinusoid densities were found in pd/uNB and tumors from patients with disseminated disease. pd/uNB also had a higher SA and, finally, high-risk patients with disseminated disease and MNA tumors had a predominance of sinusoids (Figure 3). In general, sinusoids in tumors from patients with INRG parameters associated with poor prognosis were larger, rounder, similar to convex contour and/or branched with few protrusions. MNA tumors were also associated with a regular outline, in contrast to tumors with undifferentiated histopathology, which presented an irregular outline. Larger area, greater roundness, and similar to convex contour sinusoids were also correlated with greater undifferentiation within the three NB histopathology categories. Table 4 shows the p-values and the nature of the relationship between the sinusoids and the INRG poor-prognostic factors.

**Table 4:** p-values and nature of the relationship between the sinusoids and the INRG poor-prognostic factors

Parameter	Stage: metastatic	Age: >18 months	Histopathology: (pd, uNB)	*MYCN:* MNA	Genetic profile: SCA	11q: 11qD	Risk group: high-risk
Density	0.050↑	-	0.041↑	-	-	-	-
SA	-	-	0.024↑	-	-	-	-
Rel. density	0.043↑	-	-	0.004↑	-	-	0.034↑
Rel. SA	-	-	-	-	-	-	-
Area	-	-	0.035↑, *0.009↑	0.000↑	-	-	0.001↑
Length	-	-	-	0.015↑	-	-	-
Width	0.024↑	-	0.018 ↑	0.004↑	-	-	0.001↑
Perimeter	-	-	-	-	-	-	-
Aspect	0.001↓	-	0.005↓	0.017↓	-	-	0.001↓
Roundness	0.018↓	-	*0.011↓	0.000↓	-	-	0.000↓
Per. ratio	-	-	0.016↓	0.022↑	-	-	-
Deformity	-	-	-	-	-	-	-
Shape factor	0.000↓	-	0.001↓, *0.043↓	0.000↓	-	-	0.000↓
Branching	0.017↑	0.033↑	-	0.043↑	-	-	0.000↑

SA: Stained area Rel. density: relative density, Rel. SA: relative SA, Per. ratio: perimeter ratio, -: not statistically significant, ↑/↓: higher or lower median value for the poor-prognostic group(s)

* statistically significant differences between the different degrees of differentiation within the NB histological category.

### Discontinuous post-capillaries & metarterioles are frequently found in unfavorable NB

The density of post-capillary venules & metarterioles was low in tumors from patients over 18 months of age. MNA tumors had a higher proportion of post-capillaries & metarterioles than MNNA tumors. Larger, rounder, more similar to convex contour, and post-capillaries & metarterioles with few protrusions correlated with the INRG poor-prognostic variables, in general, compared with their favorable counterparts. Nevertheless, these vessels had more protrusions in MNA tumors than in MNNA tumors. Branched post-capillaries & metarterioles were detected in MNA tumors and those from high-risk patients compared to MNNA tumors and those from non-high-risk patients. Table 5 provides the p-values and nature of the relationship between the post-capillaries & metarterioles and the INRG poor-prognostic factors.

**Table 5:** p-values and nature of the relationship between the post-capillary venules and metarterioles and the INRG poor-prognostic factors

Parameter	Stage: metastatic	Age: >18 months	Histopathology: (pd, uNB)	*MYCN:* MNA	Genetic profile: SCA	11q: 11qD	Risk group: high-risk
Density	-	0.034↓	-	-	-	-	-
SA	-	-	-	-	-	-	-
Rel. density	-	-	-	0.023↑	-	-	-
Rel. SA	-	-	-	-	-	-	-
Area	0.018↑	-	-	0.000↑	-	-	0.000↑
Length	0.033↑	-	-	-	-	-	-
Width	0.039↑	-	-	0.003↑	-	-	0.000↑
Perimeter	-	-	-	-	-	-	-
Aspect	0.044↓	-	0.013↓	0.005↓	-	-	0.000↓
Roundness	-	-	-	0.000↑	-	-	0.000↓
Per. ratio	-	-	-	-	-	-	-
Deformity	-	-	-	-	-	-	-
Shape factor	0.012↓	-	0.039↓	0.000↓	-	-	0.000↓
Branching	-	-	-	0.005↑	-	-	0.000↑

SA: Stained area Rel. density: relative density, Rel. SA: relative SA, Per. ratio: perimeter ratio, -: not statistically significant, ↑/↓: higher or lower median value for the poor-prognostic group(s).

### Blood vessels patterns are prognostic in neuroblastoma

Table 6 shows the influence of morphometric blood vessel variables in combination with the INRG prognostic factors on poor event-free survival (EFS) and overall survival (OS). Regarding sinusoids, metastatic stage and MNA had the greatest influence on poor EFS and OS followed by short length and 11q deletion (11qD) (hazard ratio (HR) 0.8, range 0.7-0.9). High sinusoid relative density correlated with poor OS between the variables short length and 11qD (HR 1.0, range 1.0-1.1). Regarding post-capillaries & metarterioles, the INRG parameters metastatic disease and undifferentiated histopathology had the greatest influence on EFS. Other significant parameters were thickness of post-capillaries & metarterioles (large width) (HR 1.4, range 1.1-1.7), then segmental chromosome aberrations (SCA) and, finally, short vessel length (HR 0.7, range 0.5-0.9).

**Table 6:** Influence of INRG prognostic factors and morphometric variables of total vascularization and blood vessel segments with value as new prognostic factors on poor EFS and/or OS of neuroblastoma patients

Variable	Wald	HR (95% CI)	P-value
Total vascularization, EFS and OS			
Non-informative			
Capillaries, EFS and OS			
Non-informative			
Sinusoids, EFS			
Metastatic stage	20.1		0.000
MNA	16.4	3.2 (1.8-5.8)	0.000
Short length	8.6	0.8 (0.7-0.9)	0.003
11qD	8.1	2.2 (1.2-3.9)	0.004
Sinusoids, OS			
MNA	24.1	5.0 (2.6-9.5)	0.000
Metastatic stage	19.9		0.000
Short length	9.8	0.8 (0.7-0.9)	0.002
High relative density	6.6	1.0 (1.0-1.1)	0.002
11qD	5.6	2.1 (1.1-4.0)	0.017
Post-capillaries & metarterioles, EFS			
Metastatic stage	22.04		0.000
uNB&pdNB	6.9		0.072
Large width	7.7	1.4 (1.1-1.7)	0.005
SCA	4.9	2.5 (1.1-5.7)	0.027
Short length	4.4	0.7 (0.5-0.9)	0.035
Post-capillaries & metarterioles, OS			
Non-informative			

EFS: Event-free survival, OS: Overall survival, MNA: MYCN amplification, 11qD: 11q deletion, u: undifferentiated NB, pd: poorly differentiated NB, SCA: Segmental chromosome aberrations.

## DISCUSSION

Several studies have revealed the importance of vascular density in the prognosis of different malignancies [10-13]. Although TMAs do not normally include areas of invasion or large blood vessels, for our study, we selected areas that better represent the tumor histology where the exchange of cells, nutrients and chemicals takes place and alters tumor cell behavior and vice versa. The application of digital pathology methods to TMA samples now allows data to be collected and translated to biological significance. Our results show that the ECM of tumors from patients with unfavorable characteristics of the INRG variables with independent prognostic value contains mostly sinusoids and a minority of capillaries. In addition, a high proportion of sinusoid vessels and the size of post-capillaries & metarterioles are related to survival, information which may be helpful to further stratify patients.

In general, few histological features of tumor vessels have been described in the literature. The few studies that exist, define highly disorganized, tortuous structures, dilated and poorly covered by pericytes, with uneven diameter, excessive branching and shunts [14-16]. Similarly, our results show that total blood vessels of tumors of patients with unfavorable-associated INRG prognostic features are larger, more branched and present a stronger deformity, compared to those from tumors of patients with favorable clinical and biological characteristics. Abnormal vascular phenotypes may be associated with the acquisition of the angiogenic capacity in cancerous tissues in response of various signals, including metabolic stress, mechanical stress, immune/inflammatory response and genetic mutations [17, 18]. Tumor vessel walls are not always formed by a homogeneous layer of endothelial cells. Instead, they may be lined with only cancer cells or a mosaic of cancer and endothelial cells, where cancer cells imitate endothelial cells in what is known as vasculogenic mimicry [19]. Cancer cells invading the vessel lumen, vessel co-option, or the apoptosis of endothelial cells which exposes underlying cancer cells could be alternative hypotheses to vessel wall mosaicism [14]. Indeed, both vascular mimicry and vessel invasion were recognized in some of the samples of our cohort. These angiogenic changes result in structurally and functionally abnormal tumor vessels. The larger and rounder capillaries of unfavorable tumors presumably correlate with a terminal network configuration (such as brain and kidney); whereas favorable tumor capillaries, which are smaller and more ovoid or bevel-cut would correlate with plexiform (such as dermis) network configurations. The anastomotic plexiform or network configuration would avoid hypoxia in the case of occlusion of vascular networks by tumor embolisms. Hypoxia enhances mutational rate, metastatic spread, and resistance to therapy, it also promotes dedifferentiation of NB and breast carcinoma cells as well as the development of stem cell-like features [20]. We hypothesize that a good plexiform capillary network would be required to maintain tissue homeostasis; nevertheless, in combination with a sinusoid-enriched ECM this could contribute to an increase in intratumoral pressure and chaos within the tumor tissue. In our cohort, tumors with sinusoid vessels that form swollen areas or cisterns, in contrast to complex sinusoidal networks, which would form longer vessels, are associated with NB patients with associated INRG poor prognostic factors.

In addition, any lack of endothelial cells in the vessel walls would be filled by the morphometric tool, thus causing an alternation in the outlines which may explain the abundant protrusions of post-capillaries & metarterioles found in MNA tumor. This anomaly would correspond to discontinuous and permeable post-capillaries & metarterioles. An increase in vessel permeability enables the extravasation of stem and immune cells, among others, and the entrance of malignant cells into the bloodstream to promote metastasis [21]. The abnormal morphology of tumor blood vessels causes a fluid pressure which has been identified as one of the culprits that impedes effective cancer treatment [22].

Our data demonstrate that blood vascularization determines the fate of the neuroblastic cells. Given the central relevance of angiogenesis in tumor development, it also represents an “Achilles' heel” that can be used for cancer therapy. As a result, multiple antiangiogenic therapeutic strategies have been developed in the last decades for many different malignancies [14]. Several direct angiogenic inhibitors of endothelial cell functions (angiostatin, endostatin and thrombospondin) and indirect anti-antiangiogenic agents that block the production or activity of pro-angiogenic molecules, such as vascular endothelial growth factor (VEGF) or platelet-derived growth factor (PDGF), have been shown to reduce tumor interstitial pressure [23-25]. Recent studies have focused on the value of normalizing tumor vasculature to improve response to conventional anticancer therapies rather than destroying tumor vessels to starve primary tumors from oxygen and induce tumor shrinkage [26]. Vessel normalization results in reduced vessel diameter, increased pericyte coverage and normalized basement membrane, with consequent recovery of normal function. This normalized tumor vasculature becomes less permeable and tortuous and leads to reduced fluid and protein extravasation into the interstitium, resulting in a decrease in tumor interstitial pressure [27, 28]. Some physical approaches to decreasing tumor ECM pressure have also been tested, including irradiation [29], hyper/hypothermic [30] or ultrasounds [31], among others [32, 33]. These strategies were made possible through the study of the physiopathology of vascularization in tumor ECM.

To summarize, NB presents different vascular segment densities, especially for capillaries and sinusoids. Furthermore, not only vessel density, but also the morphologic parameters of shape and size are differentially found in the neuroblastic histologic categories and may potentially be useful to predict survival. These morphometric variables could therefore be taken into account to enhance the pre-treatment risk stratification. Additionally, these findings, together with vascular normalization approaches, offer a new spectrum of therapeutic opportunities whose application in NB should be considered.

## MATERIALS AND METHODS

### Samples

We analyzed 19 TMA containing at least two representative cylinders of 1mm from 458 primary tumors, referred to the Spanish Reference Centre for NB Biological and Pathological studies (Department of Pathology, University of Valencia) from 1996 to 2007. Histologic and genetic studies were approved by the Spanish Society of Pediatric Hematology and Oncology (file number: 59C18ABR2002), and the European Committee (file number: 2010-021396-81), as well as by the Ethical Committee of the University of Valencia (file numbers: H1270128195640 and A1420714159483). Participants or their family members/legal guardians provided written informed consent for histological and genetic studies performed in our laboratory. Clinical data were provided by the pediatric oncologists in charge or by the Reference center for NB clinical studies.

### INRG clinical and biological data

**Figure 4:**
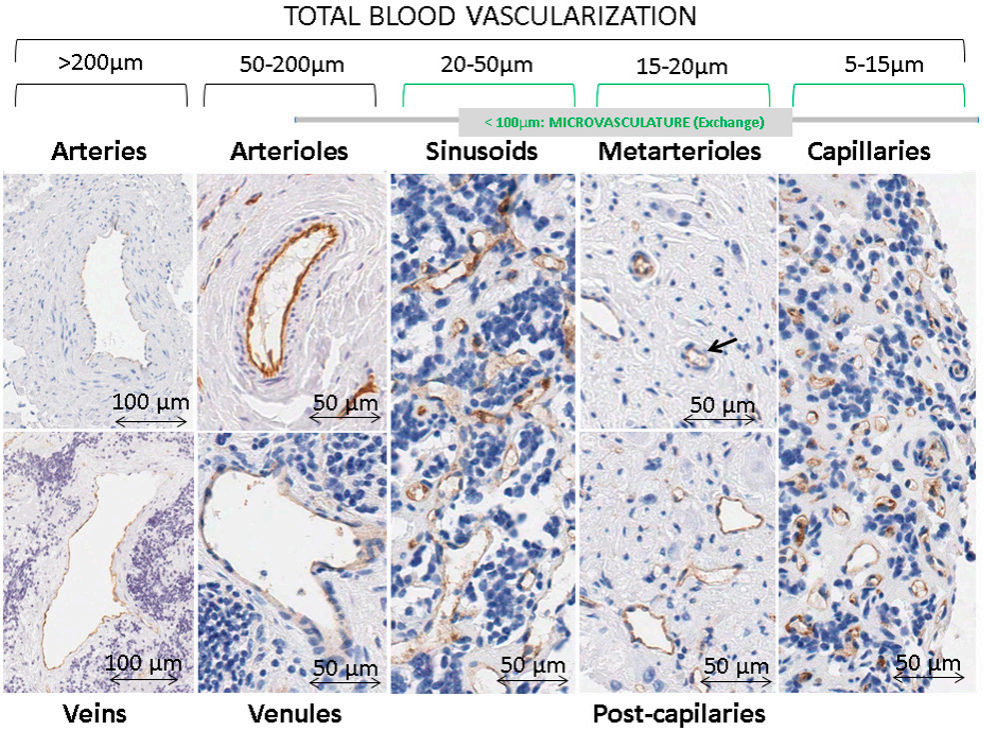
Types of blood vessels stained with immunohistochemistry anti-CD31 in neuroblastoma samples Differences in blood vessel networks can be appreciated between sinusoids and capillaries. Sinusoidal networks are shown as discontinuous capillaries with more variable forms and sizes, irregular shapes and larger caliber (around 20 to 50 μm) than an ordinary capillaries. Capillary networks are composed by vascular tubes, measuring around 5-15 μm, lined with an uninterrupted layer of endothelial cells. The intrinsic design of the TMAs avoided areas with huge vascular structures so very few samples presented venules & arterioles and veins & arteries in their ECM (1.7% and 0.1% of all present blood vessels, respectively), thus these structures were not considered.

Whole slide paraffin sections were stained with hematoxylin and eosin and IHC markers, and examined by a pathologist to select the representative areas to include in the TMA blocks and to categorize them according to the International NB Pathology Classification [34], into GN (Schwannian stroma-dominant) (n=18, 3.9%), iGNB (Schwannian stroma-rich) (n=29, 6.3%), nodular Ganglioneuroblastoma (nGNB) (composite Schwannian stroma-rich/stroma-dominant and stroma-poor) (n=4 u/pd NB nodular component, 0.9%; n=1 dNB nodular component, 0.2%) and NB (Schwannian stroma-poor) with three grades of neuroblastic differentiation: dNB (n=35, 7.6%), pdNB (n=270, 59%) and uNB (n=60, 13.1%). NB NOS samples have also been quantified (n=41, 9%). Various techniques were performed to assess the status of the MYCN oncogene: MNNA (n=379, 82.8%) *vs.* MNA (n=77, 16.8%), the integrity of the 11q23 region: non-deleted (n=321, 70.1%) *vs.* 11qD (n=77, 16.8%) and the overall genomic profile: numerical chromosomal aberrations (n=95, 20.7%) *vs.* SCA (n=223, 48.7%), following previously published European guidelines [35-39]. The combination of all the above histopathological and genetic variables with age: <18 months (n=266, 58.1%) *vs.* >18 months (n=172, 37.6%); and stage: localized 1 (n=145, 31.6%), localized 2 (n=143, 31.4%), metastatic (n=112, 24.4%) and metastatic special (n=32, 6.9%); according to the INRG classification, defined a risk group: very low (n=184, 37.6%), low (n=77, 37.6%), intermediate (n=34, 37.6%), unspecified low or intermediate (some data missing) (n=24, 5.3%) and high (n=110, 37.6%) (Figure 4) [2]. The remaining samples corresponded to unknown results.

### Histopathological analysis

All samples were independently examined by three researchers to identify common or repeating blood vessel patterns in neuroblastic cells or immune cells, or any other vascular structures of histological interest.

### Image analysis

Serial sections of 3μm were cut and immunostained with anti-CD31 (Dako, clone JC70A, 1/50) for blood vessels. The slides were scanned with a whole-slide scanner Aperio XT (Aperio technologies) at 40x magnification and tiff format was chosen. We used the previously published custom-designed tool, with minor updates, which closes open-outline vessels in order to measure morphometric parameters and properly quantify the vascular density in the different microvascular supply segments (total vasculature, capillaries, sinusoids, post-capillaries & metarterioles, venules & arterioles and veins & arteries), according to their size (Figure 4). The measurements included quantity (density, percentage of stained area (SA), relative density and relative SA for each segment) plus size (area, length, width and perimeter) and shape (aspect, roundness, perimeter ratio, deformity, shape factor and branching) (Table 1) [40]. The authors have made a huge effort in trying to understand how different values of the parameters can be reflected in real blood vessels. We have used hundreds of data corresponding to individual blood vessels of different samples, together with summaries of all blood vessels measured in tumor and control samples with different vascular patterns (liver, placenta, adrenal gland, kidney, etc…) and we have adapted the morphometric tool to be able to measure drawings with specific different morphologies. Although we consider the translation to biological significance as being quite accurate, we understand that this is sometimes difficult to see.

### Statistical methods

For statistical purposes, the histopathology variables were grouped as GN+iGNB *vs*. d(nGNB+NB) *vs*. pd/u(nGNB+NB) *vs*. other histopathologies (NB NOS). The risk variables were also been grouped as non-high risk (very low + low + intermediate) *vs*. high risk. The numerical variables derived from the morphometric analysis were related to the INRG categories using the Mann-Whitney or Kruskal-Wallis tests. The influence of the variables on survival was checked using Cox regression analysis with the stepwise forward (Wald) method. For this purpose, only samples from patients with complete information for all the variables (clinical, biological and morphometric) were considered. New morphometric parameters were related to survival individually and in combination with the INRG prognostic factors. The significance level was established at 95%.

## SUPPLEMENTARY FIGURE AND TABLE



## References

[1] Ambros PF, Ambros IM, Brodeur GM, Haber M, Khan J, Nakagawara A, Schleiermacher G, Speleman F, Spitz R, London WB, Cohn SL, Pearson AD, Maris JM (2009). International consensus for neuroblastoma molecular diagnostics: report from the International Neuroblastoma Risk Group (INRG) Biology Committee. British journal of cancer.

[2] Cohn SL, Pearson AD, London WB, Monclair T, Ambros PF, Brodeur GM, Faldum A, Hero B, Iehara T, Machin D, Mosseri V, Simon T, Garaventa A, Castel V, Matthay KK (2009). The International Neuroblastoma Risk Group (INRG) classification system: an INRG Task Force report. Journal of clinical oncology.

[3] Canete A, Navarro S, Bermudez J, Pellin A, Castel V, Llombart-Bosch A (2000). Angiogenesis in neuroblastoma: relationship to survival and other prognostic factors in a cohort of neuroblastoma patients. J Clin Oncol.

[4] Jakovljevic G, Culic S, Stepan J, Kosuta I, Seiwerth S (2011). Relationship between tumor vascularity and vascular endothelial growth factor as prognostic factors for patients with neuroblastoma. Coll Antropol.

[5] Meitar D, Crawford SE, Rademaker AW, Cohn SL (1996). Tumor angiogenesis correlates with metastatic disease, N-myc amplification, and poor outcome in human neuroblastoma. J Clin Oncol.

[6] Ozer E, Altungoz O, Unlu M, Aygun N, Tumer S, Olgun N (2007). Association of MYCN amplification and 1p deletion in neuroblastomas with high tumor vascularity. Appl Immunohistochem Mol Morphol.

[7] Peddinti R, Zeine R, Luca D, Seshadri R, Chlenski A, Cole K, Pawel B, Salwen HR, Maris JM, Cohn SL (2007). Prominent microvascular proliferation in clinically aggressive neuroblastoma. Clin Cancer Res.

[8] Ribatti D, Surico G, Vacca A, De Leonardis F, Lastilla G, Montaldo PG, Rigillo N, Ponzoni M (2001). Angiogenesis extent and expression of matrix metalloproteinase-2 and-9 correlate with progression in human neuroblastoma. Life Sci.

[9] Erdreich-Epstein A, Shimada H, Groshen S, Liu M, Metelitsa LS, Kim KS, Stins MF, Seeger RC, Durden DL (2000). Integrins alpha(v)beta3 and alpha(v)beta5 are expressed by endothelium of high-risk neuroblastoma and their inhibition is associated with increased endogenous ceramide. Cancer Res.

[10] Ullah E, Nagi AH, Lail RA (2012). Angiogenesis and mast cell density in invasive pulmonary adenocarcinoma. J Cancer Res Ther.

[11] Haldorsen IS, Stefansson I, Gruner R, Husby JA, Magnussen IJ, Werner HM, Salvesen OO, Bjorge L, Trovik J, Taxt T, Akslen LA, Salvesen HB (2014). Increased microvascular proliferation is negatively correlated to tumour blood flow and is associated with unfavourable outcome in endometrial carcinomas. Br J Cancer.

[12] Ozerdem U, Wojcik EM, Duan X, Ersahin C, Barkan GA (2013). Prognostic utility of quantitative image analysis of microvascular density in prostate cancer. Pathol Int.

[13] Barau A, Ruiz-Sauri A, Valencia G, Gomez-Mateo Mdel C, Sabater L, Ferrandez A, Llombart-Bosch A (2013). High microvessel density in pancreatic ductal adenocarcinoma is associated with high grade. Virchows Arch.

[14] Carmeliet P, Jain RK (2000). Angiogenesis in cancer and other diseases. Nature.

[15] Bergers G, Benjamin LE (2003). Tumorigenesis and the angiogenic switch. Nat Rev Cancer.

[16] Sozio F, Rossi A, Weber E, Abraham DJ, Nicholson AG, Wells AU, Renzoni EA, Sestini P (2012). Morphometric analysis of intralobular, interlobular and pleural lymphatics in normal human lung. J Anat.

[17] Gullino PM (1978). Angiogenesis and oncogenesis. J Natl Cancer Inst.

[18] Kerbel RS (2000). Tumor angiogenesis: past, present and the near future. Carcinogenesis.

[19] Folberg R, Maniotis AJ (2004). Vasculogenic mimicry. APMIS: acta pathologica, microbiologica, et immunologica Scandinavica.

[20] Pietras A, Johnsson AS, Pahlman S (2010). The HIF-2alpha-driven pseudo-hypoxic phenotype in tumor aggressiveness, differentiation, and vascularization. Curr Top Microbiol Immunol.

[21] Reymond N, d'Agua BB, Ridley AJ (2013). Crossing the endothelial barrier during metastasis. Nat Rev Cancer.

[22] Heldin CH, Rubin K, Pietras K, Ostman A (2004). High interstitial fluid pressure - an obstacle in cancer therapy. Nat Rev Cancer.

[23] Salnikov AV, Heldin NE, Stuhr LB, Wiig H, Gerber H, Reed RK, Rubin K (2006). Inhibition of carcinoma cell-derived VEGF reduces inflammatory characteristics in xenograft carcinoma. Int J Cancer.

[24] Tong RT, Boucher Y, Kozin SV, Winkler F, Hicklin DJ, Jain RK (2004). Vascular normalization by vascular endothelial growth factor receptor 2 blockade induces a pressure gradient across the vasculature and improves drug penetration in tumors. Cancer Res.

[25] Fan Y, Du W, He B, Fu F, Yuan L, Wu H, Dai W, Zhang H, Wang X, Wang J, Zhang X, Zhang Q (2013). The reduction of tumor interstitial fluid pressure by liposomal imatinib and its effect on combination therapy with liposomal doxorubicin. Biomaterials.

[26] Carmeliet P, Jain RK (2011). Principles and mechanisms of vessel normalization for cancer and other angiogenic diseases. Nat Rev Drug Discov.

[27] Goel S, Wong AH, Jain RK (2012). Vascular normalization as a therapeutic strategy for malignant and nonmalignant disease. Cold Spring Harb Perspect Med.

[28] Jain RK, Tong RT, Munn LL (2007). Effect of vascular normalization by antiangiogenic therapy on interstitial hypertension, peritumor edema, and lymphatic metastasis: insights from a mathematical model. Cancer Res.

[29] Multhoff G, Vaupel P (2012). Radiation-induced changes in microcirculation and interstitial fluid pressure affecting the delivery of macromolecules and nanotherapeutics to tumors. Front Oncol.

[30] Sen A, Capitano ML, Spernyak JA, Schueckler JT, Thomas S, Singh AK, Evans SS, Hylander BL, Repasky EA (2011). Mild elevation of body temperature reduces tumor interstitial fluid pressure and hypoxia and enhances efficacy of radiotherapy in murine tumor models. Cancer Res.

[31] Watson KD, Lai CY, Qin S, Kruse DE, Lin YC, Seo JW, Cardiff RD, Mahakian LM, Beegle J, Ingham ES, Curry FR, Reed RK, Ferrara KW (2012). Ultrasound increases nanoparticle delivery by reducing intratumoral pressure and increasing transport in epithelial and epithelial-mesenchymal transition tumors. Cancer Res.

[32] Stuhr LE, Raa A, Oyan AM, Kalland KH, Sakariassen PO, Petersen K, Bjerkvig R, Reed RK (2007). Hyperoxia retards growth and induces apoptosis, changes in vascular density and gene expression in transplanted gliomas in nude rats. J Neurooncol.

[33] Kleemann B, Loos B, Scriba TJ, Lang D, Davids LM (2014). St John's Wort (Hypericum perforatum L.) Photomedicine: Hypericin-Photodynamic Therapy Induces Metastatic Melanoma Cell Death. PLoS One.

[34] Shimada H, Ambros IM, Dehner LP, Hata J, Joshi VV, Roald B, Stram DO, Gerbing RB, Lukens JN, Matthay KK, Castleberry RP (1999). The International Neuroblastoma Pathology Classification (the Shimada system). Cancer.

[35] Lejeune M, Lopez C, Bosch R, Korzynska A, Salvado MT, Garcia-Rojo M, Neuman U, Witkowski L, Baucells J, Jaen J (2011). JPEG2000 for automated quantification of immunohistochemically stained cell nuclei: a comparative study with standard JPEG format. Virchows Archiv : an international journal of pathology.

[36] Yamamoto G, Nannya Y, Kato M, Sanada M, Levine RL, Kawamata N, Hangaishi A, Kurokawa M, Chiba S, Gilliland DG, Koeffler HP, Ogawa S (2007). Highly sensitive method for genomewide detection of allelic composition in nonpaired, primary tumor specimens by use of affymetrix single-nucleotide-polymorphism genotyping microarrays. Am J Hum Genet.

[37] Ambros IM, Benard J, Boavida M, Bown N, Caron H, Combaret V, Couturier J, Darnfors C, Delattre O, Freeman-Edward J, Gambini C, Gross N, Hattinger CM, Luegmayr A, Lunec J, Martinsson T (2003). Quality assessment of genetic markers used for therapy stratification. Journal of clinical oncology.

[38] Piqueras M, Navarro S, Canete A, Castel V, Noguera R (2011). How to minimise the effect of tumour cell content in detection of aberrant genetic markers in neuroblastoma. British journal of cancer.

[39] Piqueras M, Subramaniam MM, Navarro S, Gale N, Noguera R., Stanta G (2011). Chapter 34: Fluorescence In Situ Hybridization (FISH) on formalin-Fixed Paraffin-Embedded (FFPE) Tissue Sections. Guidelines for molecular analysis in archive tissues.

[40] Tadeo I, Piqueras M, Montaner D, Villamon E, Berbegall AP, Canete A, Navarro S, Noguera R (2014). Quantitative modeling of clinical, cellular, and extracellular matrix variables suggest prognostic indicators in cancer: a model in neuroblastoma. Pediatric research.

