# Supplementary material for: Vascular patterns provide therapeutic targets in aggressive neuroblastic tumors
Source: Oncotarget. 2016 Feb 24;7(15):19935–47. doi: 10.18632/oncotarget.7661 (PMC4991429; doi:10.18632/oncotarget.7661)
Supplement: Supplementary file 1 [file oncotarget-07-19935-s001.pdf]

SUPPLEMENTARY FIGURE AND TABLE

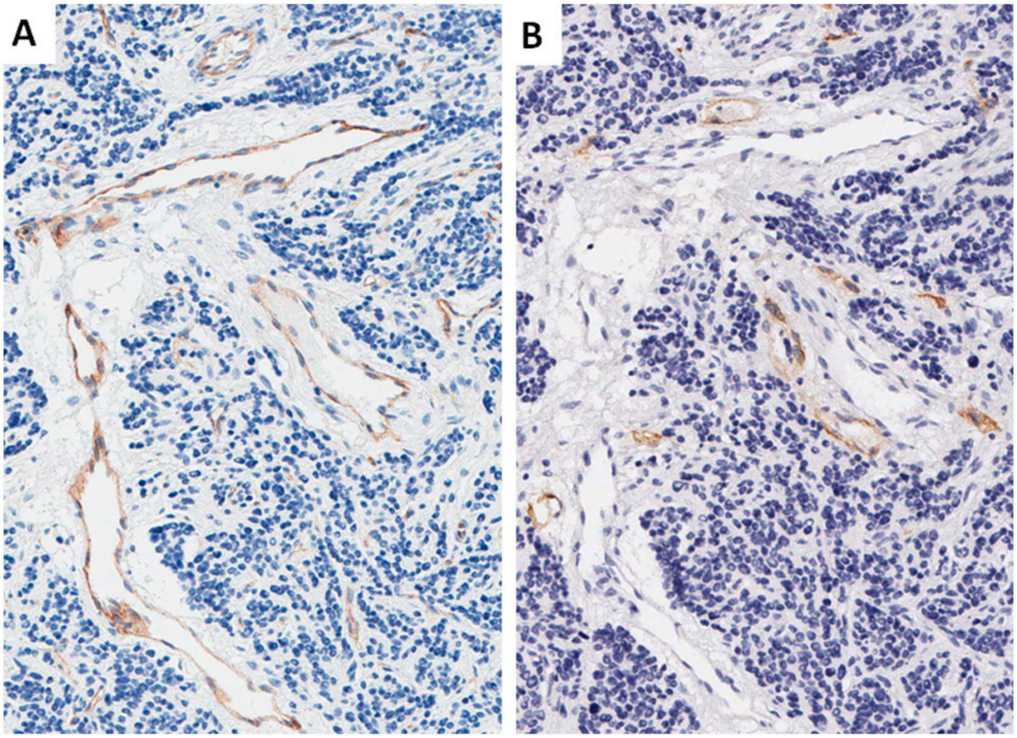

Supplementary Figure S1: Complementary stainings of A. Blood vessels by CD31 antibody and **B.** Lymph vessels by D2-40 antibody.

Supplementary Table S1: Descriptors of the total vascularization and blood vessel segments variables

Parameter	Mean	Median	Standard deviation	Minimum	Maximum
Total vascularization					
Density	161.1	102.4	177.7	0	1105
Stained area	1.7	1.2	1.9	0	15
Average area	100.9	69.6	278.9	22	4801
Length	13.9	12.5	13.1	8	239
Width	6.8	6.6	1.7	4	21
Perimeter	42.4	40	14.4	21	187
Aspect	2.2	2.1	0.2	2	4
Roundness	2.4	2.3	0.3	2	4
Perimeter ratio	0.84	0.85	0.01	0.79	0.90
Deformity	600.6	427.2	652.8	42	6198
Shape factor	2.2	0.4	21.5	0.1	373
Branching	2.7	2.7	0.2	2	4

(*Continued*)

Parameter	Mean	Median	Standard deviation	Minimum	Maximum
Blood vessel segments					
Capillaries					
Density	121.1	84.1	130.9	0	735
Stained area	0.5	0.4	0.4	0	3
Relative density	67.6	76.1	27.4	0	100
Relative stained area	32.3	30.8	21.1	0	100
Average area	30.8	31.5	5.6	18	50
Length	8.6	8.6	0.4	7	10
Width	5	6.2	0.6	4	7
Perimeter	25.7	26	1.9	20	33
Aspect	2.1	2	0.3	2	4
Roundness	1.9	1.9	0.2	2	3
Perimeter ratio	0.87	0.87	0.008	0.84	0.91
Deformity	93.6	93.5	15.1	42	155
Shape factor	0.5	0.2	1.5	0.1	26
Branching	2.4	2.4	0.1	2	3
Sinusoids					
Density	24.8	19.7	27	0	200
Stained area	0.52	0.31	0.61	0	4
Relative density	9.87	10.4	6.3	0	28
Relative stained area	24.7	27.6	13.7	0	60
Average area	196.3	191.3	57.7	84	768
Length	28.4	28.4	2.7	20	50
Width	12.8	12.8	2	7	27
Perimeter	95	94.6	13.7	64	210
Aspect	2.6	2.5	0.3	1	4
Roundness	4.1	4	0.76	2	14
Perimeter ratio	0.75	0.75	0.37	0.58	0.88
Deformity	1478.3	1456.2	398	356	3699
Shape factor	1.1	0.7	2	0.7	26
Branching	3.8	3.8	0.3	3	6
Post-capillaries & metarterioles					
Density	18.5	14.7	19	0	143
Stained area	0.18	0.12	0.19	0	1
Relative density	7.8	8.5	4.1	0	23
Relative stained area	9.8	10.8	5.7	0	37

(*Continued*)

Parameter	Mean	Median	Standard deviation	Minimum	Maximum
Average area	88	88.1	14.6	34	145
Length	17.1	17.2	0.8	16	19
Width	8.92	8.98	1.2	4	13
Perimeter	54.5	54.8	4.6	19	71
Aspect	2.3	2.2	0.4	1	6
Roundness	2.9	2.8	0.4	1	4
Perimeter ratio	0.79	0.79	0.04	0.41	0.90
Deformity	467	168.2	93	109	1004
Shape factor	0.74	0.44	1.2	0.1	13
Branching	3.26	3.29	0.3	1	4
